# Congenital Fissure of the Sternum

**Published:** 1859-06

**Authors:** Austin Flint

**Affiliations:** Professor of Clinical Medicine, etc., in the New Orleans School of Medicine


### NEW YORK MONTHLY REVIEW,

AND

### BUFFALO MEDICAL JOURNAL.

VOL. 15. JUNE, 1859. NO. 1.

### ORIGINAL COMMUNICATIONS.

ART. I.—*Congenital Fissure of the Sternum. Remarks on the Case of Mr. E. A. Groux.* By Austin Flint, M. D., Professor of Clinical Medicine, etc., in the New Orleans School of Medicine.

With the general features of the case of congenital fissure of the sternum presented in the person of Mr. Groux, most members of the medical profession in this country are sufficiently acquainted, through various publications in the medical journals. Mr. Groux has visited most of the larger cities of the Union, and afforded an opportunity for personal examination to a large number. The fissure is situated in the median line, and extends the whole length of the sternum. When the muscles of the chest are at rest, the fissure, near the upper extremity of the bone, is an inch in width; but by bringing the pectoralis muscles into strong action, the width is increased to two inches.

In this fissure is a pulsating tumor, the pulsations visible at a considerable distance, as well as perceived by the touch. "The pulsations of the tumor extend from the second to the fourth intercostal space, and from the median line to the left edge of the fissure." Above this tumor, by pressing the finger firmly on the median line, a pulsation is felt, but not seen. The feeling is that of a wave, or current. The phenomena connected with these two pulsating bodies invest this case with interest. It is also interesting as furnishing phenomena relating to the lungs and the action

WI
NE754

of certain muscles. But I propose, in the following remarks, to confine myself to the phenomena which have relation to the action of the heart.

Of the two pulsating bodies, that which is situated uppermost, and felt, not seen, is clearly a large artery. For the sake of distinction, I will call the pulsation here *arterial.* The pulsating body situated below, which is both seen and felt, is evidently some portion of the heart. This, however, is a point to be substantiated, for some distinguished observers have thought that it is the aorta. I will distinguish the pulsation here as *cardiac.* We have, then, to consider phenomena connected with, *first*, a *cardiac*, and *second*, an *arterial* pulsation, perceptible in the fissure of Mr. Groux.

### THE CARDIAC PULSATION.

The first point to be settled is, that this pulsation is cardiac—in other words, that the tumor is, in reality, a portion of the heart. The demonstration of this is as follows: The pulsation situated above this tumor is undoubtedly arterial. This is not denied by any one. Now, if the lower pulsation be also arterial, it should occur synchronously with the upper pulsation. That the two pulsations are not synchronous is apparent to the touch. Their non-synchronism is also shown by other methods : viz., by means of the sphygmoscope and the electro-megnetic machine, to be referred to presently. Other facts also show that the lower pulsating body is not arterial; but the fact which has been stated is demonstrative, and hence additional evidence is not required. If not arterial, it must be cardiac.

Assuming this tumor to be cardiac, a difference of opinion exists among the numerous persons who have examined the case, as to the portion of the heart which constitutes it. The question lies between the right auricle and the right ventricle. It is sufficiently clear from the situation of the tumor, and its relations to the point of apex-beat, that it must be a portion of either the right auricle or the right ventricle. Able observers differ as to this question. The greater number think that the pulsating body is auricular. The committee appointed by the New York Pathological Society to report on the case, consisting of Professors Dalton, Metcalfe and Peaslee, are of opinion that this tumor is a portion of the right ventricle. The committee, in their report, state the reasons for this opinion, and the distinguished names of the gentlemen who compose this committee, lend to the opinion great weight. In endeavoring to arrive at a conclusion, independently of the opinions of others, the observable phenomena which bear upon the question, are to be considered. With regard to these phenomena, the discrepancy which exists among different observers is remarkable. There is a singular want of agreement concerning points of direct and simple observation. With this contradictory testimony, one is compelled to trust to his own observations, however much he may be disposed to defer to the observations of others. It is proper for me to add, that my opportunity for examining the case of Mr. Groux was brief, being mainly confined to a single sitting.

Assuming that the cardiac pulsation is ventricular, it should occur synchronously with the apex-beat, or the latter should follow after an interval scarcely appreciable. Now the pulsation over the tumor and the impulse over the apex are not synchronous. A distinct interval is perceived by the touch. This non-synchronysm is manifested to the eye by means of the simple and beautiful instrument lately devised by Dr. J. Scott Alison, of London, called the sphygmoscope. It is also shown by the ingenious application of electro-magnetism in a machine constructed after the suggestion of Dr. J. B. Upham, of Boston. This machine is so contrived that slight movements communicated to instruments analogous to the sphygmoscope of Dr. Alison, break the electric current, and are indicated by the strokes of hammers on small bells differing in tone, so that appreciable intervals between two movements are clearly indicated to the ear.* By these three methods, viz.: the touch, the sphygmoscope and the electro-magnetic machine, the synchronism or non-synchronism of different movements or impulses is tested by evidence derived from three senses: viz., feeling, seeing and hearing. As regards the point under consideration, the fact of non-synchronism is appreciable by each of these senses, applied in the modes just stated. If the cardiac pulsation be ventricular, then it must be admitted that a distinctly appreciable interval exists between the contraction of the right ventricle and the apex-beat; in other words, that the two ventricles do not contract in perfect unison, the apex-beat being considered as representing the contraction of the left ventricle. The length of the interval between the pulsation of the tumor and the apex-beat has a bearing on the point just stated. Dr. Upham, by means of the application of electro-magnetism, in connection with the chronographic apparatus at the Cambridge observatory, ascertained that this interval is 38-1000 of the time occupied by the beat of the heart.*

* For an account of this machine and observations made with it in the case of Mr. Groux, see paper by Dr. Upham, entitled "Some Account of the Recent Experiments made in connection with the case of M. Groux," read before the Boston Society for medical improvement, and communicated to the Boston Medical and Surgical Journal.

* *Vide* paper by Dr. Upham, for an account of the novel and interesting experiments with reference to this question.

Assuming the cardiac pulsation to be auricular, it should not occur synchronously with the apex-beat. The contraction of the auricles, as shown by vivisections, precedes, by a brief interval, the ventricular contraction. This is precisely the relation which apparently exists between the pulsation of the cardiac tumor, in the case of Mr. Groux, and the systolic impulse over the apex.

An experiment performed by Mr. Groux, with reference to the point under consideration, consists in attaching two small feathers, by means of wax, to the chest, one directly over the cardiac tumor, and the other at the lower extremity of the fissure. The feather placed over the tumor presents a rotary motion, and that placed below is motionless, in tranquil breathing. But on taking a deep inspiration, by which the heart is moved downward and forward (as shown by the late experiments on the cadaver, by Dr. Da Costa, of Philadelphia), the lower, as well as the upper feather, presents a rotary motion. This experiment is, however, not of much value, for it is difficult to determine the existence or absence of synchronism. The two feathers evidently do not move in the same direction, but this is not sufficient to prove that the movement of each is not due to the systole of the right ventricle.

Another experiment performed by Mr. Groux is perhaps open to fallacy. On placing one sphygmoscope over the point of apex-beat, and another above, over the right ventricle, two movements are produced, which are synchronous. This is intended to prove that the contraction of the right ventricle and the apex-beat are absolutely simultaneous, and hence that the pulsation of the cardiac tumor cannot be ventricular. The liability to fallacy is this: both sphygmoscopes may, possibly, be affected by the apex-beat; in other words, the movement communicated to the upper sphygmoscope, as well as to the lower, may be due to the impulsion of the apex of the heart against the thoracic walls.

Again: assuming that the cardiac pulsation in the fissure is ventricular, it should be synchronous with the arterial pulsation above; at all events, a distinct interval should not separate the two pulsations. The touch, however, suffices to show that they are not synchronous, but that a distinct interval occurs between them. On the other hand, the arterial pulsation and the apex-beat are synchronous, as determined by the touch.

The foregoing facts appear to demonstrate that the cardiac tumor is a portion of the right auricle, and not of the right ventricle. Another method of investigation with reference to this question, consists in determining the relations of the two pulsations and the apex-beat to the heart-sounds. The apex-beat is shown by the sphygmoscope to be synchronous with the first or systolic sound. The pulsation of the cardiac tumor cannot be synchronous with this sound, but I had not an opportunity of studying, satisfactorily, its precise relation to the first and second sounds, nor of observing the arterial pulsation in this point of view.

The Committee of the New York Pathological Society base their opinion that the cardiac pulsation in the fissure proceeds from the right ventricle, *first*, on the situation of the tumor; *second*, on the character of the pulsations; and *third*, on the enlargement of the tumor produced by a prolonged expiration.* Assuming that the tumor is a portion of the right auricle, the heart is not in its normal situation. That it is situated higher than usual, is shown by the apex-beat being in the fourth, instead of the fifth intercostal space, the posture being sitting or standing. Its position must also be more vertical, and the base removed farther to the left than in well-formed chests. It is not unreasonable to suppose that, under the circumstances, this abnormal situation exists. Careful percussion might perhaps settle this point. The pulsations of the tumor are described as "wavy and peristaltic, running from above downward, and from right to left." As opposed to these, it is stated that "the contractions of the appendix auriculæ (the only part of the right auricle which could present anteriorly) are different in character, and are directed from the point of the appendix toward the body of the auricle, more posteriorly and toward the right." It is to be remarked, with reference to this point, that a depression exists within the limits of the fissure, and inspection extends somewhat below the anterior surface of the heart. The enlargement of the cardiac tumor produced by a forced expiration is remarkable. It is caused by the mechanical obstruction to the pulmonary circulation. It is evident, and vivisections, as well as examinations after death, show that the right auricle, as well as the right ventricle, becomes distended under these circumstances, so that the enlargement of the tumor cannot be considered as proving that it is not auricular.

So far as the cardiac pulsation is concerned, in the case of Mr. Groux, the question of its source is chiefly interesting as a matter of curiosity. In view of the discrepancy of opinion with regard to this question, although an individual observer may arrive at a conclusion which is satisfactory to himself, he is not warranted in assuming this conclusion to be indubitably correct. The phenomena, therefore, connected with this pulsation, cannot become of much real value to science until its source is determined by an autopsical examination, which, in the case of Mr. Groux, it is to be hoped, may be deferred for many years to come. It is a clin-

* For this report, *vide* the American Medical Monthly, January, 1859.

ical problem, in the case, to determine what portion of the heart constitutes this tumor; when, to be really useful in its application to physiology, there should be no room for discussion on this point.

Were the source of the pulsations established beyond all doubt, the opportunity for studying its relations to the movements and sounds of the heart would be more valuable than they can at present bé made to the satisfaction of all minds.

On applying the stethoscope over the cardiac tumor, the two sounds of the heart are distinctly heard, the second sound being more intense than the first. The latter fact is interesting as showing the predominance of the second sound over the valvular element of the first sound. On auscultation over the right auricle, I was unable to satisfy myself as to an auricular sound, the existence of which is supposed, by some observers, to be proved by this case. My examination, however, was not sufficiently directed to this point to enable me to arrive at the conclusion that an auricular sound is not present.

### ARTERIAL PULSATION.

The pulsation of the tumor which is seen in the fissure, being demonstrated to be cardiac, the pulsating body felt on deep pressure with the finger just above this tumor, is undoubtedly the aorta. The pulsation is wavy, and gives the idea of a current of liquid propelled through an elastic tube of considerable size. It is an undulatory rather than a pulsative movement. As compared by the touch, it occurs synchronously with the apex-beat of the heart. I did not perceive a movement consecutive to the apex-beat or the ventricular systole, and representing the force which gives rise to the expansion of the semilunar valves and the second or diastolic sound of the heart; but my attention was perhaps not sufficiently directed to this point.

With the sphygmoscope placed over the aorta, a movement is represented to the eye. This movement is not synchronous, but alternates with the apex-beat as represented by another sphygmoscope placed at the same time over the apex of the heart. This fact is very clearly and beautifully shown. The aortic motion as felt by the finger, and as seen by the eye through the medium of the sphygmoscope, is evidently not the same; in the one case it is synchronous with the first sound of the heart, and in the other case it coïncides with the second sound. The movement seen with the sphygmoscope must be due to the recoil of the arterial coats after the distension caused by the current of blood propelled into the arteries by the left ventricle. It represents, in other words, the force which expands the semilunar valves, and produces the aortic second sound of the heart. With the electro-magnetic apparatus, this is beautifully illustrated. The arterial motion, synchronous with the apex-beat, and that which coïncides with the second sound, are, from time to time, signalized each by a stroke on one of the two bells, the two strokes following each other in quick succession, the second stroke representing the arterial recoil, being the louder of the two. Assuming that there is here no error of observation, these experiments are highly interesting as illustrating the force which causes the expansion of the semilunar valves, and the production of the second sound. Comparing the relative force of the two motions, viz., the motion synchronous with the apex-beat, and that coïncident with the second sound, as represented by the sphygmoscope and the two bells, it is intelligible that an aortic regurgitant murmur should be as loud as it is sometimes observed to be in cases of insufficiency of the aortic valves. It is also intelligible that in aortic lesions with insufficiency, an aortic regurgitant murmur may be present without an aortic direct murmur—a fact which clinical observation shows to be of not unfrequent occurrence. Moreover, the force of this recoil explains the intensity of the aortic second sound of the heart, this sound being more widely diffused than the first sound.

With the stethoscope (Cammann's) placed over the aorta, the two heart-sounds are heard; the first relatively distant and less intense, but varying in intensity with the action of the heart; the second near the ear, quite intense, presenting the characters of the aortic second sound as heard usually in the right second intercostal space, except that its intensity is greater. A soft systolic bellows murmur is also heard, probably produced by the pressure of the instrument upon the artery.

By taking a deep inspiration, and making a strong effort of expiration with the glottis closed, Mr. Groux is able to arrest the arterial pulsation of the wrist. This is probably owing to the pressure of the expanded lung on the subclavian artery. The ability thus to arrest the pulse is not peculiar to Mr. Groux. I find that I can do it, and I presume it can be generally done.

